# Characteristics of matched international medical graduates in a private Saudi university and factors predicting number of interviews

**DOI:** 10.1097/MD.0000000000049752

**Published:** 2026-07-17

**Authors:** Khaled S.M. Elshaer, Faisal Sannaa, Tarek Ziad Arabi, Abdulrahman Comert, Ahmed El Shaer, Wassel Sannaa, Nader Ashraf, Belal Nedal Sabbah, Wael Alkattan, Khaled Alkattan, Akef Obeidat, Emadeddin Raddaoui, Abderrahman Ouban

**Affiliations:** a College of Medicine, Alfaisal University, Riyadh, Saudi Arabia; b Department of Cardiovascular Medicine, University of Michigan, Ann Arbor, MI; c Department of Medicine, University at Buffalo-Catholic Health System, Buffalo, NY.

**Keywords:** clinical electives, international medical graduates, residency match, USMLE, visa status

## Abstract

International medical graduates (IMGs) from the Middle East face unique challenges in the US residency match process. Alfaisal University (AU), a private institution in Riyadh, Saudi Arabia, has consistently achieved match rates above the global IMG average, warranting investigation into the characteristics and predictors of success among its graduates. This study examines matched AU graduates’ profiles and identifies factors associated with the number of residency interviews received, to inform strategies for future IMG applicants. A cross-sectional, survey-based study was conducted among AU graduates who matched into US residency programs between September 2023 and September 2024. The survey, modeled after the NRMP questionnaire, captured demographics, academic metrics, clinical and research experiences, and match outcomes. Statistical analysis included univariate and multivariate linear regression to determine predictors of interview count. A total of 92 matched AU graduates participated. Most were first-time applicants (89.6%) and directly matched (96.7%). The mean Step 1 and Step 2 clinical knowledge scores were 247.1 ± 10.5 and 251.2 ± 12.8, respectively. Participants reported an average of 6.1 research experiences, 5.3 work experiences, and 2.6 electives. In multivariate analysis, the number of US clinical electives (β: 1.10, *P* = .02) and non-visa-requiring status (β: 5.53, *P* = .001) were significantly associated with a higher number of interviews. AU IMGs demonstrated competitive academic and research profiles relative to their US and international peers. Key predictors of interview success were completion of US clinical electives and not requiring a visa.

## 1. Introduction

International medical graduates (IMGs) are physicians who received their medical degree and training from a medical school located outside the country where they intend to practice or do their residency. IMGs seeking to continue their training in the United States can further be classified into 2 categories: those with US citizenship (US IMGs) and those without (non-US IMGs).^[1]^

Alfaisal University (AU) is a private university in Riyadh, Saudi Arabia with a large multiethnic student population. In 2024, AU college of medicine achieved a match rate of 64.3%, the highest in Saudi Arabia. Over the past 6 years, the university’s graduates’ match rate into residency programs has exceeded the global average for international medical schools.^[2]^ Despite the publication of NRMP data on an annual database, there remains limited data available on predictors of matching for IMGs, especially in the Middle East.

This study aims to analyze the characteristics of AU IMGs who matched and the predictors of receiving an interview among applicants. The findings will help identify key factors that played a significant role in AU IMGs securing residency interviews. Our results will contribute to the broader understanding of IMG success in the US residency match process.

## 2. Materials and methods

The study takes place in AU’s College of Medicine: a privately funded institute in Riyadh, Saudi Arabia. Each year, a significant number of Alfaisal graduates are successful in matching to residency programs in the United States. Matched AU applicants across the US were asked to participate in a survey-based questionnaire to examine factors that lead to their success in securing a residency position.

The survey was constructed based on the National Residency Matching Program (NRMP) questionnaire, covering essential topics such as program selection factors, program ranking factors, the number of applications submitted, and the number of interviews received. The survey domains aligned with NRMP categories including demographics, examination scores (Step 1, Step 2 clinical knowledge [CK], Step 3), research and work experiences, program applications, and match outcomes. Additional input regarding survey development was provided by matched AU applicants and the college’s director of academic affairs. The survey encompassed 32 items: 7 items assessing demographics, 8 items assessing examination scores and certifications, eleven items assessing application details and experiences, and 6 items assessing the matching process and outcomes.

Demographic questions cover foundational information such as graduation year, cGPA, and visa requirements (J-1/H-1B/US citizen/Green card). Items covering examination scores investigate applicants’ United States Medical Licensing Examination (USMLE) Step 1 and 2 CK scores. Applicants who took USMLE Step 3 were also asked about their score and whether it was released before or after programs were able to review Eelectronic Residency Application Service applications.

To ensure validity, questions were structured similarly to those of the NRMP applicant survey. Additionally, the final questionnaire passed approval by the local Office of Academic Affairs at our university. The survey was created and distributed using SurveyMonkey. The survey platform required completion of all mandatory fields before submission; however, participants could skip optional items. Missing data were excluded from analysis on a per-variable basis (complete case analysis).

The study population consists of Alfaisal graduates who have applied to US residency programs. Graduates who matched locally and outside of the US were excluded from the study population.

This study utilized a purposive sampling technique. A message containing an introductory paragraph that details the study’s aim and affirms participant anonymity as well as the liberty to withdraw or decline to respond along with the survey link was circulated via the university’s institutional email system to the target population. Additionally, the survey was sent to matched applicants via text messages. Of the 230 AU graduates eligible to participate (those who applied to US residency programs during the 2023–2024 cycle), 92 completed the survey, yielding a response rate of 40%. This response rate is consistent with published surveys in medical education research, where rates of 40% to 60% are commonly observed.^[3]^ Responses were collected using the survey starting from September 2023 until September 2024.

Categorical data were described as counts. For quantitative data, the mean ± standard deviation were calculated. Statistical analysis was performed using GraphPad Prism 9 version 9.4.1 (GraphPad Software, San Diego). *T*-tests and chi-squares were used to compare between 2 groups. Variance inflation factors (VIF) were calculated to assess multicollinearity among predictor variables; all VIF values were below 1.4 (maximum VIF = 1.38), indicating no substantial collinearity (VIF < 5 threshold).^[4]^ Linear regression models were used to determine factors associated with the number of interviews. Linear regression was selected given the sufficiently large count outcomes (mean = 9.05 interviews). Regression assumptions were assessed: residuals demonstrated approximate normality (Shapiro–Wilk *P* = .14); however, the Breusch–Pagan test indicated potential heteroscedasticity (*P* = .007). Factors with a *P*-value < 0.1 were included in a multivariable model. Statistical significance was set at *P* < .05.

In compliance with the provisions of the Saudi Law of Ethics of Research on Living Creatures and regulations, and under the guidelines of the National Committee of Bioethics, ethics approval was acquired from the institutional review board (Reference IRB-20158). Informed consent was obtained from all graduates.

## 3. Results

A total of 92 participants from Alfaisal University who matched into a US residency program were included in the study. Out of those who responded, 69 (89.6%) were first-time applicants and 8 (10.4%) were not. The majority of the applicants (96.7%) directly matched into a program; however, only 2 applicants made it through the Supplemental Offer and Acceptance Program (“SOAP”).

When it comes to the USMLE, all of the participants took Step 1 and Step 2 CK, but 68.1% took Step 2 CS and Step 3. For those who took USMLE Step 1 prior to it being pass-fail (54.5%), the mean score was 247.09 ± 10.54; 100% of respondents who took the exam after it became pass/fail passed the exam. Most (93%) of those who took Step 2 CS passed the exam on the first attempt. The mean scores for Step 2 CK and Step 3 were 251.24 ± 12.82 and 233.76 ± 14.81, respectively.

**Figure 1.**
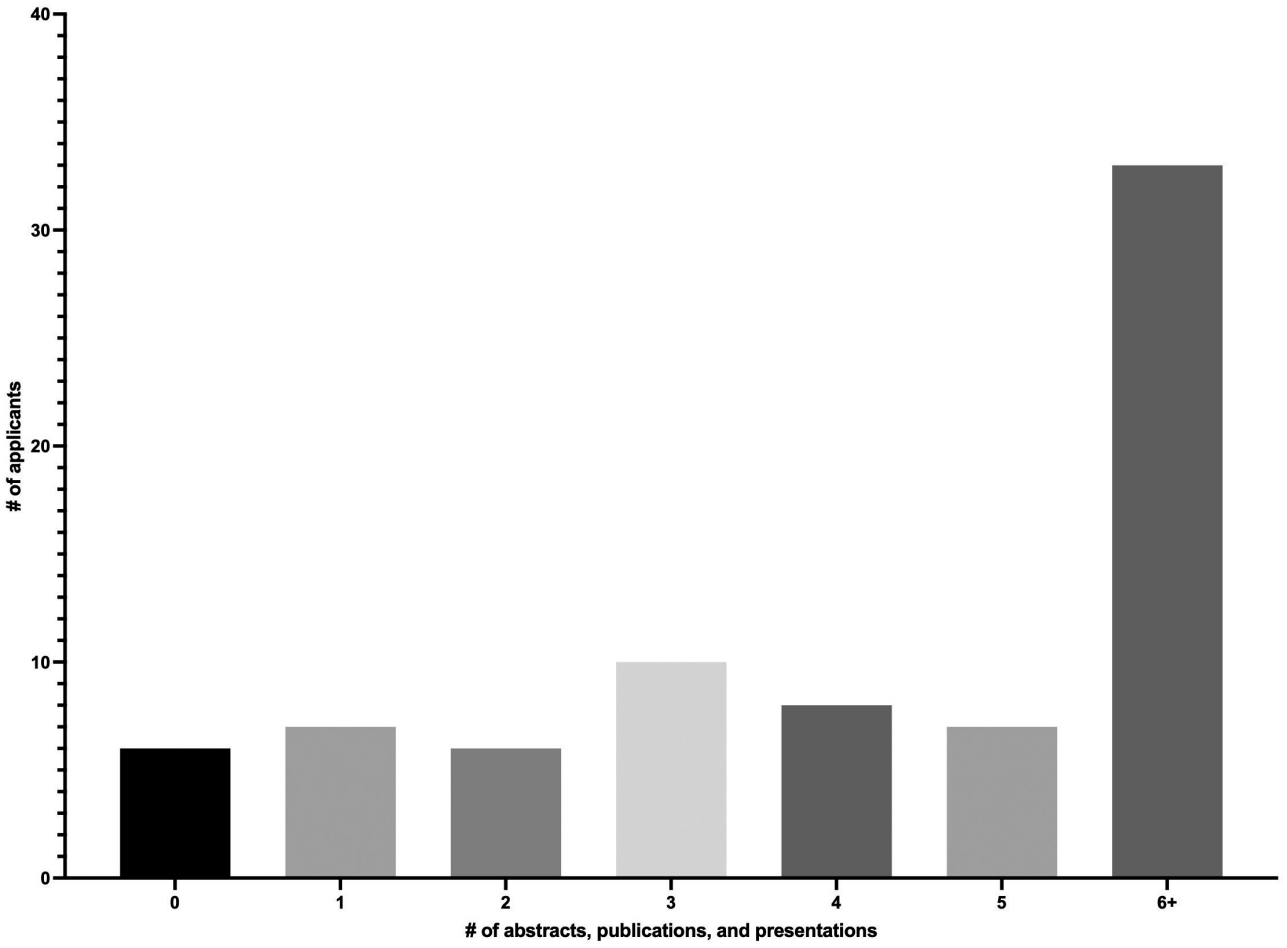
The number of match applicants achieving a certain number of work experiences at Alfaisal University.

**Figure 2.**
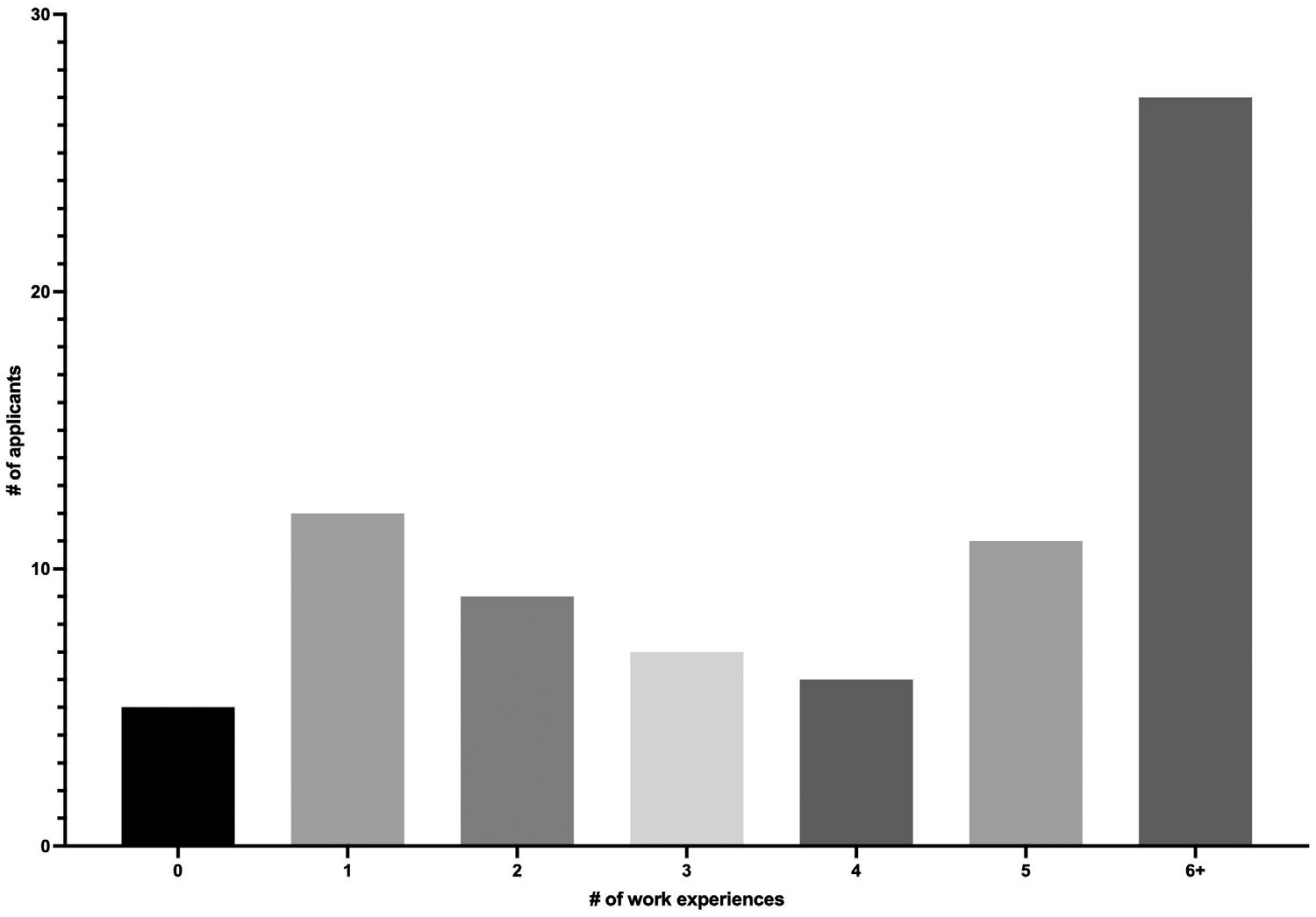
The number of match applicants achieving a certain number of publications at Alfaisal University.

Participants reported a mean of 6.10 ± 8.26 research experiences (Fig. 1), 5.27 ± 5.22 work experiences (Fig. 2), 7.62 ± 9.34 publications, and 2.56 ± 1.55 electives. Moreover, they ranked a mean of 8.38 ± 5.19 programs and received 9.05 ± 5.54 interviews. Most of the participants (60%) applied to internal medicine, followed by 11.7% who applied to multiple specialties and 6.7% to each of pediatrics and neurology.

In the univariate analysis, the cGPA (β: 7.35, 95% CI: 2.77–11.92, *P* = .002), Step 2 CK score (β: 0.05, 95% CI: 0.02–0.23, *P* = .02), number of work experiences (β: 0.47, 95% CI: 0.06–0.89, *P* = .03), and number of electives (β: 1.81, 95% CI: 1.01–2.61, *P* < .0001) were statistically significant. A non-visa requiring status was not statistically significant (*P*-value = .09).

However, when assessed using multivariate analysis (Table 1), the number of electives retained significance (β: 1.10, 95% CI: 0.19–2.01, *P* = .02), and the requirement of non-visa status became significant (β: 5.53, 95% CI: 2.44–8.63, *P* = .001). The other parameters, such as cGPA, Step 2 CK score, and number of work experiences, did not maintain their significance.

**Table 1:** Predictors of the number of interviews received.

Paramater	Univariate			Multivariate		
	β	95% CI	*P*-value	β	95% CI	*P*-value
cGPA	7.348	2.773–11.92	.0021	3.555	−3.109 to 10.22	.2834
Step 1 score^*^	0.09909	−0.07116 to 0.2693	.2474	–		
Step 2 CK score	0.0235	0.01712–0.2276	.0235	0.09210	−0.05112 to 0.2353	.1981
Date of receiving Step 3 score						
After December 31st	Reference			–		
After programs begin reviewing applications	0.4061	−3.458 to 4.270	.8332			
Before programs begin reviewing applications	2.626	−5.362 to 5.228	.9799			
Not ECFMG certified	0.05091	−3.673 to 3.775	.9783			
Non-visa requiring	2.483	−0.4284 to 5.393	.0932	5.535	2.441–8.629	**.0011**
Applied multiple times to the match	0.2672	−4.225 to 4.759	.9057	–		
Number of research experiences	0.05016	−0.2432 to 0.3435	.7334			
Number of research publications, abstracts, and presentations	0.2083	−0.01364 to 0.4303	.0653	−0.06622	−0.3569 to 0.2244	.6439
Number of work experiences	0.4742	0.05861–0.8898	.0260	0.3243	−0.2950 to 0.9435	.2921
Number of electives	1.810	1.007–2.612	<.0001	1.102	0.1943–2.009	**.0192**
Number of observerships	−1.051	−2.447 to 0.3444	.1362	–		
Specialty applied to						
Internal medicine	Reference					
Pediatrics	−2.528	−8.347 to 3.291	.3872	–		
Family medicine	−7.194	−13.83 to −0.5598	.0341	−2.379	−9.063 to 4.306	.4716
Multiple	0.7579	−3.803 to 5.319	.7400	–		
Anesthesiology	−0.5278	−11.72 to 10.67	.9250			
Neurology	0.2222	−5.597 to 6.041	.9392			
General surgery	−1.028	−9.252 to 7.196	.8030			
Emergency medicine	0.8056	−5.829 to 7.440	.8084			

The bold values highlight parameters which retained significance after multivariate regression analysis.

cGPA = cumulative grade point average, CI = confidence interval, CK = clinical knowledge, ECFMG = educational commission for foreign medical graduates.

* For those who took the exam before pass/fail change.

When it comes to specialties, in the univariate analysis, only family medicine was negatively associated with the number of interviews compared to internal medicine. Nevertheless, when the multivariate model was used, this association did not persist.

## 4. Discussion

This study provides a comprehensive analysis of the characteristics and predictors of interview success among AU IMGs in the US residency match. Our findings highlight AU graduates’ competitive performance relative to other IMGs, emphasizing the critical role of research productivity, and clinical exposure in securing residency interviews.

Multivariate analysis identified 2 significant predictors of receiving interview invitations: the number of US clinical electives completed and non-visa-requiring status. These results align with prior evidence underscoring the importance of US clinical experience for IMGs, as it demonstrates familiarity with the healthcare system and mitigates program directors’ concerns about cultural adaptability.^[5]^ Non-visa-requiring applicants (e.g., US citizens/permanent residents) faced fewer barriers, consistent with reports that visa constraints disproportionately hinder non-US IMGs.^[6,7]^ Notably, while univariate analysis suggested associations between interview count and Step 2 CK scores, cGPA, and work experiences, these variables did not retain statistical significance in the multivariate model. Only 2 factors remained independently associated with interview count: the number of US clinical electives and non-visa-requiring status. This finding suggests that after adjusting for confounders, US clinical exposure and immigration status may be more influential than academic metrics in determining interview invitations.

AU graduates demonstrated competitive academic metrics compared to both IMGs and US graduates. Matched AU applicants reported a mean Step 1 score of 247.1 (SD 10.5) ± 10.54, which is comparable to previously reported IMG averages. According to the 2024 NRMP Charting Outcomes report, matched non-US IMGs in internal medicine had a mean Step 2 CK score of 248.^[8]^ AU IMGs had a mean Step 2 CK score of 251.2, comparable to matched US MD seniors who had a mean Step 2 CK score of 250.4.^[9]^ These scores reflect AU’s rigorous academic preparation, particularly in basic sciences assessed by Step 1, which historically served as a key differentiator for IMGs.^[10]^ Furthermore, the transition of Step 1 to pass/fail scoring has shifted emphasis to Step 2 CK and research, intensifying pressure on IMGs to excel in these areas.^[11,12]^ AU graduates’ strong Step 2 CK performance positions them favorably in this new landscape.

AU graduates reported a mean of 6.1 (SD 8.3) research experiences. For comparison, US MD seniors who matched in 2024 reported a mean of 3.8 research experiences according to NRMP data.^[8]^ However, direct comparisons should be interpreted cautiously given differences in reporting methodology and potential variation in how research experiences are defined across institutions.

Several limitations warrant consideration. First, reliance on self-reported data introduces potential recall bias, particularly among earlier graduates. Second, social desirability bias may have influenced responses regarding academic metrics and research productivity. Third, the response rate of 40% and exclusion of unmatched applicants may introduce selection bias, as respondents may disproportionately represent higher-performing graduates who successfully matched, limiting generalizability to broader IMG populations. Fourth, the modest sample size (n = 92) relative to the number of predictors examined raises concerns about potential overfitting in the multivariate model. Fifth, the Breusch-Pagan test indicated potential heteroscedasticity in the regression model, which may affect the precision of coefficient standard errors; future studies with larger samples should consider robust standard error estimation. Sixth, the impact of visa status may vary across specialties; however, our sample size precluded specialty-specific subgroup analyses. Finally, the retrospective design limits our ability to assess the impact of recent changes, including the transition of Step 1 to pass/fail scoring or other post-pandemic adjustments in residency selection.

Prospective studies should explore longitudinal trends in AU graduate performance, particularly post-Step 1 scoring changes. Comparisons with other Middle Eastern institutions could identify region-specific strategies for IMG success. Expanding datasets to include qualitative insights from program directors may further elucidate the weight of nonacademic factors, such as mentorship and institutional reputation.

## 5. Conclusion

Alfaisal University IMGs perform competitively in the US match through a combination of academic rigor, research engagement, and clinical exposure at King Faisal Specialist Hospital and Research Centre. As residency selection evolves, sustained emphasis on Step 2 CK, clinical electives, and visa-neutral pathways will be critical for IMGs worldwide. These findings underscore the importance of institutional support in navigating the complex US residency landscape.

## Author contributions

**Conceptualization:** Ahmed El Shaer, Khaled S.M. Elshaer, Wassel Sannaa, Faisal Sannaa, Abdulrahman Comert, Abderrahman Ouban.

**Data curation:** Khaled S.M. Elshaer, Faisal Sannaa, Tarek Ziad Arabi, Abdulrahman Comert, Wassel Sannaa, Nader Ashraf, Belal Nedal Sabbah, Wael Alkattan, Khaled Alkattan, Akef Obeidat, Emadeddin Raddaoui.

**Formal analysis:** Tarek Ziad Arabi, Ahmed El Shaer.

**Project administration:** Ahmed El Shaer, Abderrahman Ouban.

**Supervision:** Abderrahman Ouban.

**Writing – original draft:** Khaled S.M. Elshaer, Faisal Sannaa, Tarek Ziad Arabi, Abdulrahman Comert, Ahmed El Shaer, Wassel Sannaa, Nader Ashraf, Belal Nedal Sabbah, Wael Alkattan, Khaled Alkattan, Akef Obeidat, Emadeddin Raddaoui.

**Writing – review & editing:** Abderrahman Ouban.
